# Supplementary material for: Depletion of kinesin motor KIF20A to target cell fate control suppresses medulloblastoma tumour growth
Source: Commun Biol. 2021 May 11;4:552. doi: 10.1038/s42003-021-02075-4 (PMC8113472; doi:10.1038/s42003-021-02075-4)
Supplement: Supplementary file 2 — Supplementary Information [file 42003_2021_2075_MOESM2_ESM.pdf]

Supplementary Information

Depletion of kinesin motor KIF20A to target cell fate control suppresses medulloblastoma tumour growth

Qiu et al.

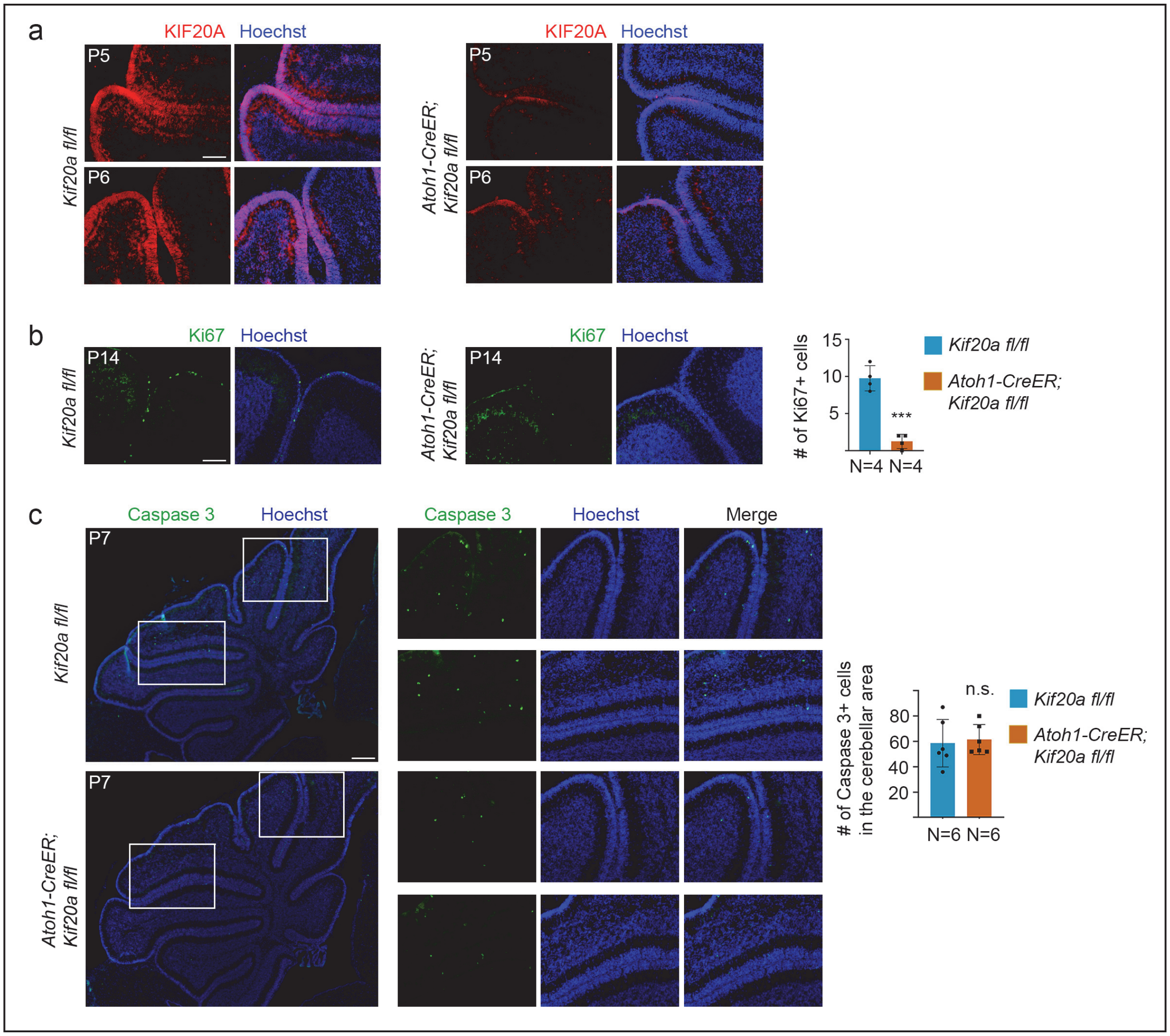

Supplementary Figure 1

Analyses of proliferation and apoptosis status in the *Kif20a* knockout cerebellum

a. Staining of KIF20A in P5 and P6 control and Kif20a knockout cerebellums. Tamoxifen was given to pups at P4. Scale bar represents 50μm.

b. Staining of proliferation marker Ki67 in P14 control and Kif20a knockout cerebellums. Tamoxifen was given to pups at P4. Data are mean ± S.D. ***P<0.001 (student's t-test).

c. Knockout of *Kif20a* did not cause noticeable change of apoptosis level marked by cleaved caspase 3 in the mutant cerebellum (P7). Scale bar represents 50μm. Data are mean ± S.D. n.s. not significant

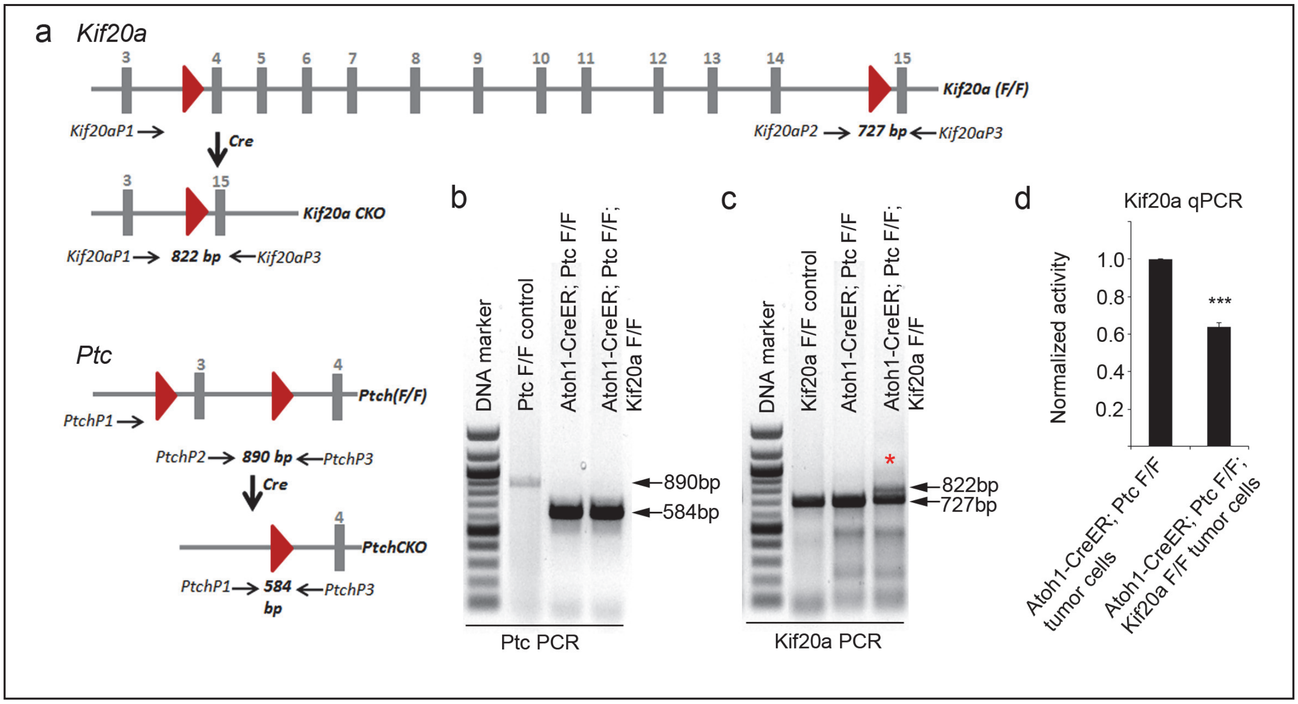

Supplementary Figure 2.

Generation of tumor cell lines from *Ptc* single and *Ptc/Kif20a* double knockout mice

a. Schematic illustration of PCR strategies for genotyping *Kif20a* and *Ptc* knockout in mouse tumor cells.

b and c. Primers of P1, P2 and P3 were combined for PCR on genomic DNAs. Tumor cells from both the single (*Atoh1-CreER; Ptc*$^{fl/fl}$) and double (*Atoh1-CreER; Ptc*$^{fl/fl}$*; Kif20a*$^{fl/fl}$) knockout mice showed homozygous deletion of *Ptc*. However, tumor cells from the double knockout mice were found to be heterozygous for *Kif20a*. *indicates the lane showing heterozygous bands of *Kif20a* alleles.

d. qPCR on RNAs isolated from tumor cells derived from the *Ptc* and *Kif20a* double knockout mice showed reduced level of *Kif20a* mRNA. ***$P<0.001$ (Student's t-test).

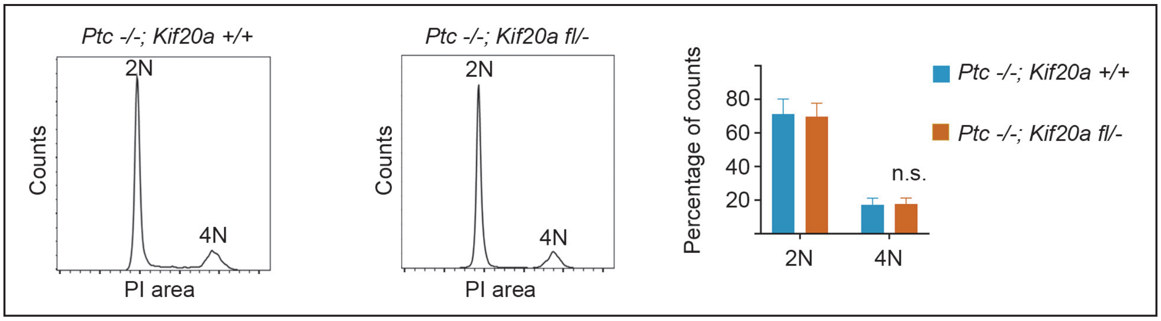

Supplementary Figure 3

FACS-based cell cycle analysis of SHH-MB tumor cell lines

Tumor cells derived from the *Ptc* single and *Ptc/Kif20a* double knockout mice were labeled with propidium iodide and their DNA profiles were examined by flow cytometry. PI, propidium iodide; 2N, cells in G1 phase; 4N, cells in G2/M phase or bi-nucleated cells. n.s. not significant.

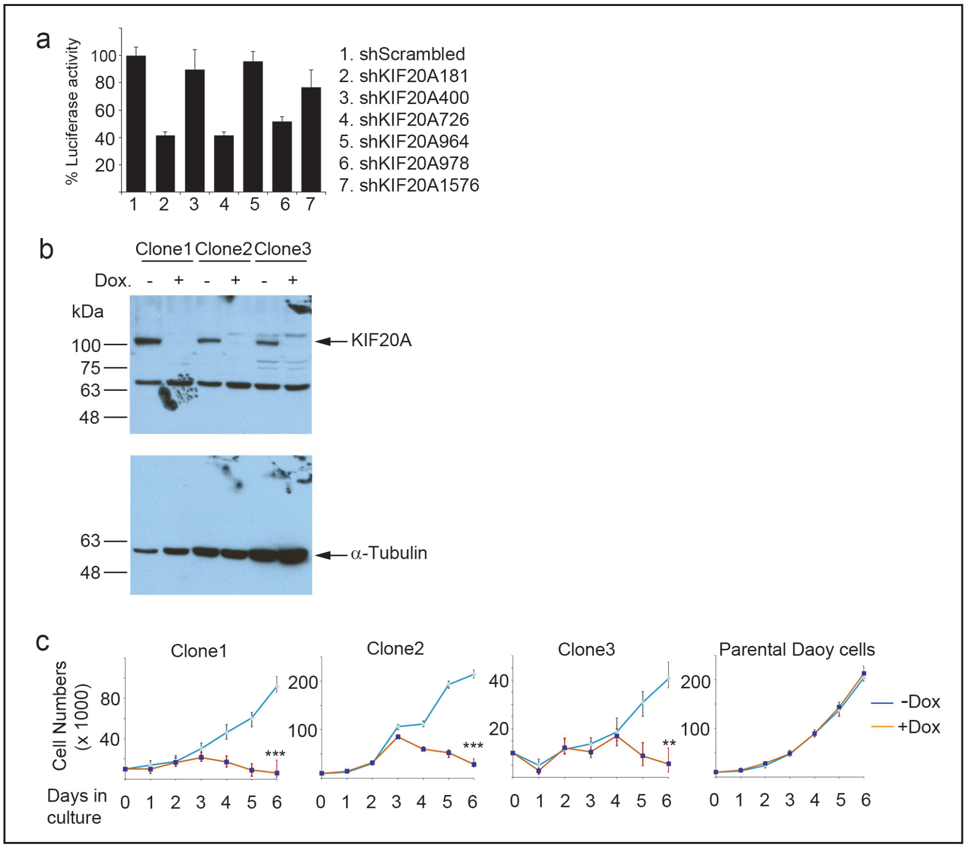

Supplementary Figure 4

Generation of human KIF20A shRNAs and stable Daoy cell lines

a. Screen of shRNAs by luciferase assay. Candidate shRNAs in pNUT vector were first screened with a cDNA target cloned in psi-CHECK vector in transfected HEK293 cells, using a dual luciferase reporter assay and their cDNA target in psi-CHECK Vector (Promega). Six candidate shKIF20As were tested with this assay. shKIF20A726 was further used to isolate stable clones of Daoy cells. Data are mean ± S.D.

b. Three Daoy cell lines stably integrated with Tet-on inducible shKIF20A726 expression cassette were established. In each cell line, doxycycline (Dox) treatment led to reduction of KIF20A expression. α-Tubulin was used as a loading control.

c. Inducible expression of shKIF20A726 (+Dox) in each cell line could inhibit cell proliferation. As a technical control, parental Daoy cells showed similar growth property with or without doxycycline treatment. Data are mean ± S.D. **P<0.01, ***P<0.001 (two-way ANOVA).
