# Supplementary material for: Depletion of kinesin motor KIF20A to target cell fate control suppresses medulloblastoma tumour growth
Source: Commun Biol. 2021 May 11;4:552. doi: 10.1038/s42003-021-02075-4 (PMC8113472; doi:10.1038/s42003-021-02075-4)
Supplement: Supplementary file 3 — Descriptions of Additional Supplementary Files [file 42003_2021_2075_MOESM3_ESM.pdf]

Description of Additional Supplementary Files

File name: Supplementary Data

Description: All source data underlying the graphs and charts presented in the main figures.
